# Perceived racism-based police violence and substance use among black and hispanic emerging adults: Evidence from a national sample

**DOI:** 10.1016/j.dadr.2025.100388

**Published:** 2025-10-15

**Authors:** Robert O. Motley, Eric Williamson, Melissa McTernan, Sara Beeler, Christopher P. Salas-Wright

**Affiliations:** a Boston College School of Social Work, USA; b Boston College Lynch School of Education and Human Development, USA; c University of Illinois Chicago, Jane Addams College of Social Work, USA

**Keywords:** Racism, Police violence, Substance use, Black or African American emerging adults, Hispanic emerging adults

## Abstract

**Purpose:**

Racism-based police violence (RPV) is an emerging public health concern. However, limited research has examined the relationship between RPV exposure and substance use, particularly among Black or African American and Hispanic emerging adults aged 18–29 years. This study assessed associations between lifetime RPV exposure across three domains (direct victimization, witnessing in person, and media-based exposure) and past 30-day and 12-month alcohol and cannabis use in this population.

**Methods:**

A cross-sectional survey was conducted from August to October 2023 using a national nonprobability internet sample recruited via Qualtrics. The analytic sample included 936 Black or African American (48 %) and Hispanic (52 %) emerging adults. Negative binomial and logistic regression models were used to examine associations.

**Results:**

Higher levels of RPV exposure were significantly associated with increased alcohol and cannabis use. RPV–Victim exposure was the strongest predictor of alcohol use, including past 30-day (IRR = 1.02, 95 % CI: 1.00–1.05) and 12-month (OR = 1.04, 95 % CI: 1.01–1.07) use. RPV–Witness exposure was most strongly associated with cannabis use, including past 30-day (IRR = 1.02, 95 % CI: 1.00–1.03) and 12-month (OR = 1.03, 95 % CI: 1.01–1.06) use. A dose-response pattern was observed across increasing levels of RPV exposure.

**Conclusions:**

RPV exposure is a significant correlate of alcohol and cannabis use among Black or African American and Hispanic emerging adults. Findings suggest that perceived racialized policing is a significant risk factor for substance use and warrants further investigation as a potential structural determinant, highlighting it as an important target for public health interventions.

## Highlights

• RPV exposure is linked to increased alcohol and cannabis use among emerging adults.
• RPV–Victim exposure most strongly predicts alcohol use across timeframes.
• RPV-Witness exposure is the strongest predictor of cannabis use.
• A dose-response pattern is observed across all RPV exposure types.
• Findings highlight RPV as a potential structural determinant of substance use disparities.

## 1 Introduction

Racism remains a pervasive determinant of health, disproportionately affecting Black or African American and Hispanic emerging adults (ages 18–29) in the United States (Andrade et al., 2021, Artiga et al., 2023, Bailey et al., 2017, Williams et al., 2019). Recognizing its profound health consequences, the American Public Health Association (2020) has declared racism a public health crisis, underscoring its role in exacerbating substance use (cannabis, and alcohol) outcomes among Black or African American and Hispanic emerging adults (Carliner et al., 2016, Gibbons et al., 2010, Otiniano Verissimo et al., 2014). One manifestation of structural racism is perceived racism-based police violence (RPV), defined as actual or threatened physical, psychological, or verbal harm by law enforcement officers that an individual perceives as racially motivated (Motley and Baidoo, 2023). RPV exposure can occur through direct (as a victim or in-person witness) or indirect (through media, such as television or online content) exposure (Motley et al., 2023). For many Black or African American and Hispanic emerging adults, RPV exposure is not an isolated event, but a recurring reality embedded within a broader system of racialized policing, shaping daily experiences and perceptions of safety (Chaney and Robertson, 2013; Hoggard & Lutchman, 2024; Motley and Baidoo, 2023; Motley and Joe, 2023; Solis et al., 2009; Wilson et al., 2021). Despite increasing recognition of racism as a public health concern, research examining RPV exposure and its association with cannabis and alcohol use remains limited.

Recent national data indicate that cannabis and alcohol remain the most used substances among adults in the United States, with notable increases in use observed among emerging adults (Substance Abuse and Mental Health Services Administration, 2024). In 2023, 36.5 % of emerging adults aged 18–25 reported using cannabis in the past year, the highest rate among all age groups, followed by emerging adults aged 26–29 (29.3 %; Substance Abuse and Mental Health Services Administration, 2024). Rates were particularly elevated among Black or African American (24.5 %) and Hispanic (18.2 %) emerging adults. Similarly, alcohol use remained prevalent, with 49.6 % of emerging adults aged 18–25 and 51.9 % of those aged 26 or older reporting past-month alcohol use (Substance Abuse and Mental Health Services Administration, 2024). Binge drinking was reported by 28.7 % of those aged 18–25. Notably, 42.5 % of Black or African American and 41.2 % of Hispanic emerging adults reported alcohol use in the past month.

Substance use, particularly cannabis and alcohol, is increasingly recognized as a health behavior outcome of traumatic experiences, functioning as a means of coping with stress, anger, fear, and other negative emotions arising from such events (D'Amico et al., 2022, Ebrahimi et al., 2024, Hawn et al., 2020, Johnson et al., 2025). For example, a recent study of Black or African American male emerging adults in Baltimore (n = 100) found that both alcohol and cannabis were used to cope with cumulative violent experiences, including child maltreatment and family, neighborhood, and interpersonal violence (Marineau et al., 2025).

RPV exposure is a potentially traumatic event for Black or African American and Hispanic emerging adults (English et al., 2017, Findling et al., 2019, Motley et al., 2025, Solis et al., 2009) that may be associated with substance use as a means of coping with the stress and emotional toll of such experiences (Lazarus and Folkman, 1984). To our knowledge, only one published study has directly examined the relationship between RPV exposure and substance use. Motley and colleagues (2022) investigated this association among 300 Black or African American emerging adult college students in St. Louis, Missouri, focusing specifically on cannabis use. Indirect RPV exposure, particularly through media, was significantly associated with increased cannabis use among Black or African American males, suggesting that vicarious experiences of RPV may contribute to substance use as a coping mechanism. However, the study was geographically restricted to a single metropolitan area and included only Black or African American college students, excluding the experiences of Hispanic emerging adults. In addition, the analysis was limited to cannabis use, thereby overlooking alcohol, the most widely used psychoactive substance in the United States and one closely linked with cannabis use (Hai et al., 2022, Salas-Wright et al., 2021, Substance Abuse and Mental Health Services Administration, 2023).

The present study addresses these gaps by utilizing a national sample of both Black or African American and Hispanic emerging adults, broadening the scope of RPV exposure to include three distinct forms (victimization, witnessing in-person, and media exposure), and examining the two most used psychoactive substances, while adjusting for other non-police involved perceived racism-based experiences and psychological distress. By doing so, this study offers a more comprehensive understanding of the health behavior consequences of RPV exposure across ethnic groups and multiple exposure pathways.

## 2 Method

### 2.1 Procedures and Participants

The institutional review board at Boston College granted the current study procedures ethical approval. A total of 1147 potential participants were recruited through Qualtrics, which employs a panel aggregation approach capable of reaching over 7 million residents in the U.S. (Qualtrics, 2014). Each potential participant received an electronic invitation containing a hyperlink to the survey, an explanation of the study's purpose, and an informed consent notification. Participant consent was implied when respondents clicked on the survey hyperlink. Each participant received remuneration in the form of a $20 gift card. This study adhered to the Checklist for Reporting Results of Internet E-Surveys (CHERRIES) reporting guideline for conducting web-based surveys using nonprobability internet panels (Eysenbach, 2004) and Strengthening the Reporting of Observational Studies in Epidemiology-Survey (STROBE-S) guideline for reporting cross-sectional findings (Bennett et al., 2010).

Data was collected from August through October 2023 as part of a cross-sectional national survey. The survey assessed self-reported experiences of RPV across three types of exposure (victimization, witnessing in-person, and media), cannabis and alcohol use, sociodemographic characteristics, and non-police involved racism-based experiences. At the conclusion of the survey, all participants were provided with a debriefing statement and a list of national mental health and crisis resources (e.g., 988 Suicide and Crisis Lifeline, Crisis Text Line, SAMHSA National Helpline, the Trevor Project, Trans Lifeline). This information was included to mitigate potential distress from survey content and to ensure participants had access to immediate support if needed.

Black or African American and Hispanic individuals were recruited nationwide using quota sampling procedures designed to approximate 2020 U.S. Census demographics for gender and ethnicity among Black or African American and Hispanic emerging adults ages 18–29 within a ± 10 % margin. A predetermined goal of 1000 completed surveys was set, and once the target sample size was reached within each demographic stratum, no additional participants were invited to complete the survey. This approach enhanced representation on the key variables of gender and ethnicity but, as a nonprobability sample, does not ensure full representativeness on other sociodemographic characteristics such as urban/rural residence or education levels. Participants who did not meet inclusion criteria or complete the survey were excluded from subgroup quotas and final analyses.

### 2.2 Measures

*Substance Use Outcomes*. Cannabis use without a physician’s orders was assessed using a measure from the PhenX Toolkit (Hamilton et al., 2011). Individuals reported their frequency of cannabis use over the past 12 months using an 11-point Likert scale ranging from 1 = 1–20 drinks to 11 = 201 or more times, and the number of days they used cannabis during the past 30 days. Alcohol use was assessed using a measure from the PhenX Toolkit (Hamilton et al., 2011). Individuals reported their frequency of drinking over the past 12 months using an 11-point Likert scale ranging from 1 = 1–20 drinks to 11 = 201 or more drinks, and the number of days they consumed alcohol during the past 30 days.

*Lifetime Exposure to Racism-Based Police Violence*. The Exposure to Racism-Based Police Violence Measure (RPV; Motley et al., 2025) comprises three distinct scales used to assess the frequency of lifetime RPV exposure using a numeric scale ranging from 0 =  never to 8 =  almost every day. The 7-item RPV-Victim scale assesses the frequency of direct RPV exposure as a victim. Examples of survey items include “I have been unarmed and had police use their weapons (e.g., Taser, pepper spray, baton, firearm) on me, and I believed it was because of my race or ethnic group.” The 6-item RPV-Witness scale evaluates the frequency of witnessing an RPV event in person. Example of survey items include “I have witnessed in-person police verbally assault an individual by using racial slurs (e.g., Spic, wetback, beaner, refi, coon, monkey, slave, boy, the “N” word) toward them, and I believed it was because of the individual’s race or ethnic group.” Lastly, the 6-item RPV-Media scale measures the frequency of exposure to videos of RPV via media (television, social media, internet). Examples of survey items include “I have seen a video of a real-life event where an individual was being attacked by a police canine (excluding the airport), and I believed it was because of the individual’s race or ethnic group.” Results from the validation study for the RPV exposure scales revealed that the final three-factor model demonstrated excellent fit indices (RMSEA =.06; CFI =.96; TLI =.95; SRMR =.05) and evidence of configural, metric, and scalar invariance across gender and ethnicity among Black or African American and Hispanic emerging adults (Motley et al., 2025), supporting the scales’ construct validity. In addition, the scales also demonstrated one-week test–retest reliability ranging from moderate to excellent (RPV-Victim r = .91, RPV-Witness r = .68, RPV-Media r = .44; Motley et al., 2025), supporting their temporal stability. Results for this sample demonstrate good to excellent internal reliability: RPV-Victim α = 0.891, RPV-Witness α = 0.932, and RPV-Media α = 0.946.

*Sociodemographic Characteristics*. For the present study, participants self-reported their ethnic identity (Black or African American, or Hispanic), gender identity (male, female, transgender, or other), age, geographic region (west, southwest, Midwest, northeast, southeast, or Puerto Rico), nativity (born in the U.S. or born outside the U.S.), income during the previous 12-months (less than $10,000, $10,000-$19,999, $20,000-$29,999, $30,000-$39,999, $40,000-$49,999, $50,000-$59,999, $60,000-$69,999, $70,000-$79,999, $80,000-$89,999, $90,000-$99,999, $100,000 or more), and employment status during the previous 12-months (unemployed, part-time, or full-time). Given that the majority (86.9 %) of participants had self-reported incomes of less than $70,000, we collapsed the income variable into four categories (1 = Less than $10k, 2 = $10k–$39,999, 3 = $40k–$69,999, 4 = $70k or more).

*Other Racism-based Events.* Exposure to non-police involved racism-based events was measured using the 9-item Everyday Discrimination Scale (EDS; Williams et al., 1997). The EDS is a widely used measure that captures the frequency of various forms of racial discrimination in individuals’ day-to-day lives (e.g., "You are treated with less courtesy than other people") on a 6-point Likert scale ranging from 0 =  never to 5 =  almost every day. The EDS has demonstrated good reliability and validity across diverse populations (Williams et al., 1997) and has been found to be liked with substance use (Clark et al., 2015, Kim et al., 2014). Scores in this sample showed good reliability (Cronbach α =.94).

*Psychological Distress*. Kessler Psychological Distress Scale (K10; Kessler et al., 2002) was used to measure participants' levels of psychological distress over the past 30 days on a 5-point Likert scale ranging from 1 =  none of the time to 5 =  all of the time. Total scores range from 10 to 50, with higher scores indicating greater psychological distress. K10 has shown strong internal consistency and discriminant validity in diverse community samples (Easton et al., 2017).

### 2.3 Statistical Analysis

All analyses for this study were conducted using R statistical software (v. 4.3; R Core Team, 2023). Bivariate associations were assessed using *t* tests and ANOVA. We employed several regression approaches based on the distribution and nature of our outcome variables. Model selection was guided by AIC statistics, with lower values indicating better fit (Burnham, Anderson, 2004). For past 30-day cannabis use, as well as past 30-day alcohol use, negative binomial regression was selected over Poisson regression due to lower AIC statistics and evidence of overdispersion (Cameron, Trivedi, 2013). Past 12-month cannabis use, and past 12-month alcohol use were analyzed using ordinal logistic regression. Effect size was measured using incidence rate ratios or odds ratios, depending on the type of outcome. Unadjusted effect sizes were generated from binomial regression models, and adjusted effect sizes were generated from multivariate models with ethnicity, gender, region, nationality, income, employment, EDS scores, and psychological distress scores as covariates. All hypothesis tests were 2-tailed and considered to be significant at an α < .05 (Detailed descriptions of missing data patterns and model diagnostics, including multicollinearity checks, are provided in Appendix A: Supplementary Methodology).

## 3 Results

### 3.1 Racism-Based Police Violence Exposure by Sociodemographic and Psychosocial Characteristics

Table 1 presents lifetime RPV exposure across three dimensions—victimization (RPV-Victim), witnessing in person (RPV-Witness), and media exposure (RPV-Media)—by sociodemographic characteristics, EDS, psychological distress, and substance use outcomes. Significant ethnic group differences were observed for both RPV-Witness (*t*(934) = 2.41, *p* = .016) and RPV-Media (*t*(934) = 2.80, *p* = .005), with Black or African American participants reporting higher mean exposure scores than Hispanic participants (RPV-Witness: *M* = 6.09, *SD* = 8.90 vs. *M* = 4.79, *SD* = 7.60; RPV-Media: *M* = 16.56, *SD* = 13.62 vs. *M* = 14.14, *SD* = 12.81).

**Table 1:** Lifetime Prevalence of Exposure to Racism-based Police Violence by Sociodemographic and Psychosocial Characteristics of the Study Population.

		RPV – Victim (n = 936)		RPV – Witness (n = 936)		RPV – Media (n = 936)	
	N (%) of respondents	M (SD)	F or *t*	M (SD)	F or *t*	M (SD)	F or *t*
Total	936 (100 %)	2.52 (5.42)	-	5.42 (8.2)	-	15.3 (13.2)	-
Ethnicity							
Black or African American	450 (48 %)	2.85 (5.96)	t(934) = 1.80^a^	6.09 (8.9)	t(934) = 2.41^a^⁎	16.56 (13.62)	t(934) = 2.80^a^⁎⁎
Hispanic	486 (52 %)	2.21 (4.85)		4.79 (7.6)		14.14 (12.81)	
Gender							
Female	526 (56.2 %)	2.48 (5.61)	F(3, 932) = .268^b^	5.24 (8.2)	F(3, 932) = .536^b^	13.9 (12.7)	F(3, 932) = 5.34^b^⁎⁎
Male	381 (40.7 %)	2.58 (5.13)		5.55 (8.2)		16.9 (13.7)	
Transgender	16 (1.71 %)	3.06 (7.5)		7.69 (9.5)		22.3 (12.7)	
Other	12 (1.28 %)	1.33 (2.27)		6 (8.7)		14.8 (12.6)	
Region							
West	162 (17.3 %)	1.62 (2.95)	F(5, 930) = 1.85^b^	4.81 (7.6)	. F(5, 930) = 926^b^	16.2 (14.2)	F(5, 930) = 1.196^b^
Puerto Rico	44 (4.70 %)	2.16 (3.59)		5.45 (7.1)		18.1 (13.5)	
Northeast	138 (14.7 %)	3.43 (6.92)		6.49 (9.9)		15.8 (13.1)	
Midwest	183 (19.5 %)	2.71 (5.52)		5.67 (8.1)		16.1 (13.3)	
Southeast	259 (27.6 %)	2.65 (6.1)		5.48 (8.6)		14.3 (12.6)	
Southwest	150 (16 %)	2.28 (4.86)		4.66 (7.1)		14.1 (13.0)	
Born in the U.S.							
Yes	858 (91.6 %)	2.58 (5.48)	t(934) = 1.23^a^	5.61 (8.2)	t(934) = 2.37^a^⁎	15.8 (13.2)	t(934) = 3.91^a^⁎⁎⁎
No	78 (8.33 %)	1.79 (4.67)		3.29 (7.7)		9.73 (11.4)	
Income							
Less than $10,000	246 (26.2 %)	1.72 (4.27)	F(3, 932) = 8.37^b^⁎⁎⁎	4.8 (9.08)	F(3, 932) = 2.64^b^⁎	14.1 (13.8)	. F(3, 932) = 993^b^
$10k-$39,999	344 (36.7 %)	2.08 (4.25)		5.01 (7.24)		15.6 (13.0)	
$40k-$69,999	210 (22.4 %)	2.98 (5.69)		5.73 (7.81)		15.4 (13.3)	
$70k or more	136 (14.5 %)	4.35 (8.3)		7.07 (9.62)		16.4 (12.4)	
Employment							
Unemployed	263 (28.1 %)	1.57 (3.86)	F(2, 933) = 8.09^b^⁎⁎⁎	4.27 (8.11)	F(2, 933) = 6.4^b^⁎⁎	13.7 (13.4)	F(2, 933) = 4.69^b^⁎⁎
Part time	259 (27.6 %)	2.32 (5.24)		4.93 (7.85)		14.5 (12.6)	
Full time	413 (44.1 %)	3.25 (6.23)		6.47 (8.5)		16.7 (13.3)	
Everyday Discrimination							
Low Discrimination	332 (35.5 %)	0.55 (1.53)	*F*(2, 933) = 68.44^b^⁎⁎⁎	1.79 (4.34)	*F*(2, 933) = 88.86^b^⁎⁎⁎	9.81 (11.21)	*F*(2, 933) = 51.53^b^⁎⁎⁎
Medium Discrimination	306 (32.7 %)	2.04 (3.89)		5.05 (7.23)		17.19 (12.55)	
High Discrimination	298 (31.8 %)	5.2 (7.91)		9.84 (10.3)		19.49 (14)	
Psychological Distress							
Low Distress	338 (36.1 %)	1.17 (3.41)	*F*(2, 933) = 26.38^b^⁎⁎⁎	3 (6.21)	*F*(2, 933) = 38.94^b^⁎⁎⁎	11.01 (12.24)	*F*(2, 933) = 47.58^b^⁎⁎⁎
Medium Distress	309 (33 %)	2.39 (4.24)		5.09 (6.75)		14.81 (11.72)	
High Distress	289 (30.9 %)	4.23 (7.58)		8.6 (10.54)		20.85 (13.97)	

Note. RPV =  racism-based police violence.

^⁎^p < .05, ⁎⁎ p < .01, ⁎⁎⁎ p < .001.

a *t* statistic from *t*-test of differences in RPV scale scores across both levels of nationality.

b F-statistic from one-way ANOVA testing for differences in RPV scale scores across levels of the demographic variable.

RPV-Media exposure differed significantly by gender (*F*(3, 932) = 5.34, *p* = .001), with transgender participants reporting the highest exposure (*M* = 22.3, *SD* = 12.7), followed by male (*M* = 16.9, *SD* = 13.7) and female participants (*M* = 13.9, *SD* = 12.7). Participants born in the United States reported significantly higher RPV-Witness (*t*(934) = 2.37, *p* = .018) and RPV-Media (*t*(934) = 3.91, *p* < .001) exposure compared with those born outside the U.S. (RPV-Witness: *M* = 5.61, *SD* = 8.29 vs. *M* = 3.29, *SD* = 7.78; RPV-Media: *M* = 15.8, *SD* = 13.2 vs. *M* = 9.73, *SD* = 11.4).

Significant differences in RPV-Victim exposure were found by income level, with individuals earning $70,000 or more reporting the highest mean scores (F(3, 932) = 8.37, *p* < .001; *M* = 4.35, *SD* = 8.30). This group also reported elevated RPV-Witness exposure (F(3, 932) = 2.64, *p* = .048; *M* = 7.07, *SD* = 9.62). Employment status was also significant across all RPV domains, with full-time workers reporting higher RPV-Victim (F(2, 933) = 8.09, *p* < .001; *M* = 3.25, *SD* = 6.23), RPV-Witness (F(2, 933) = 6.40, *p* = .002; *M* = 6.47, *SD* = 8.50), and RPV-Media exposure (F(2, 933) = 4.69, *p* = .009; *M* = 16.7, *SD* = 13.3) than part-time or unemployed participants.

Levels of EDS differed significantly across all RPV exposure domains. Participants in the high EDS group reported substantially higher scores on RPV-Victim (F(2, 933) = 68.44, *p* < .001; *M* = 5.20, *SD* = 7.91), RPV-Witness (F(2, 933) = 88.86, *p* < .001; *M* = 9.84, *SD* = 10.3), and RPV-Media (F(2, 933) = 51.53, *p* < .001; *M* = 19.49, *SD* = 14.0), compared to those reporting low EDS. Similar patterns were observed for psychological distress. Participants with high distress reported significantly greater RPV-Victim (F(2, 933) = 26.38, *p* < .001; *M* = 4.23, *SD* = 7.58), RPV-Witness (F(2, 933) = 38.94, *p* < .001; *M* = 8.60, *SD* = 10.54), and RPV-Media exposure (F(2, 933) = 47.58, *p* < .001; *M* = 20.85, *SD* = 13.97) than those with low distress.

**Ethnic Differences in Substance Use Outcomes Among Emerging Adults**

**Fig. 1:**
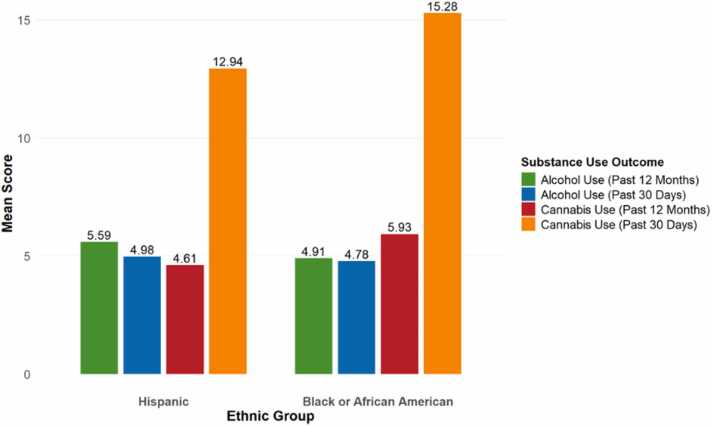
Mean Substance Use Outcomes by Ethnic Group.

Fig. 1 displays the mean average of substance use outcomes by ethnic group. Black or African American participants reported slightly higher mean scores for past 30-day cannabis use (*M* = 2.45) and past 12-month cannabis use (*M* = 3.03) relative to Hispanic participants (*M* = 2.22 and *M* = 2.85, respectively). Conversely, Hispanic emerging adults reported higher average past 30-day alcohol use (*M* = 3.43) and past 12-month alcohol use (*M* = 3.93) compared to their Black or African American peers, who reported lower means across both indicators (*M* = 2.49 and *M* = 3.13, respectively). These findings suggest differential patterns of cannabis and alcohol use by ethnicity among emerging adults, with Black or African American participants reporting slightly greater cannabis use and Hispanic participants showing greater alcohol use across the measured timeframes.

### 3.2 Association of Substance Use Outcomes with Racism-based Police Violence Exposures

**Fig. 2:**
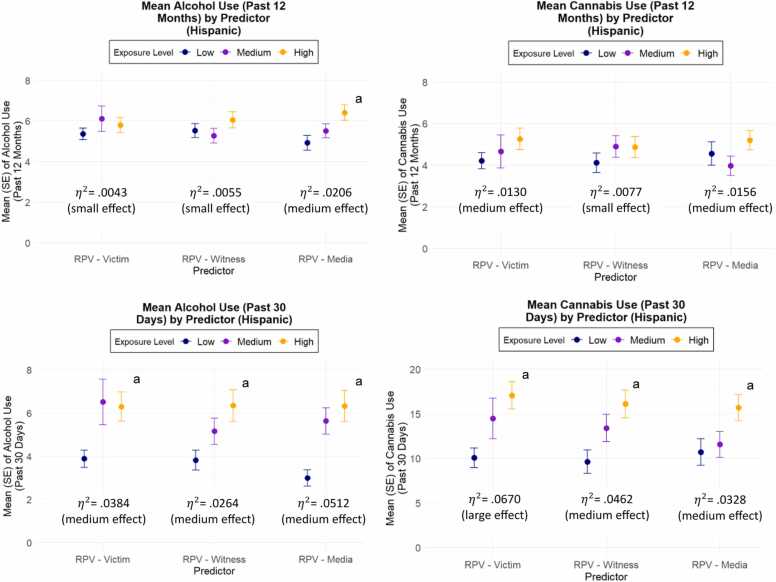
Substance Use Outcomes by Type of Lifetime Exposure to Racism-Based Police Violence (Hispanic).

**Fig. 3:**
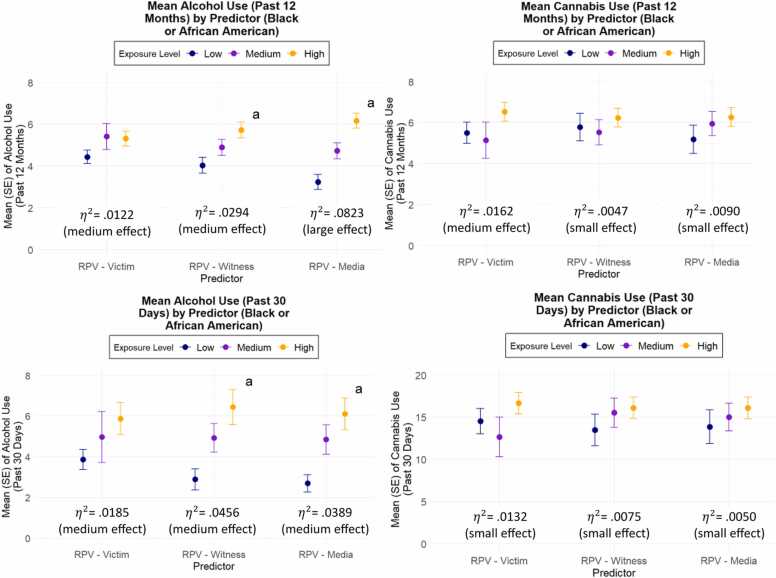
Substance Use Outcomes by Type of Lifetime Exposure to Racism-Based Police Violence (Black or African American). Note. RPV =  racism-based police violence. ^a^ Significant ANOVA result (p < .05).

Fig. 2, Fig. 3 display the prevalence of substance use outcomes stratified by terciles (low, medium, high) of lifetime RPV exposure among Black or African American and Hispanic participants, respectively. Across both groups, higher levels of RPV exposure were generally associated with increased alcohol consumption, with the most pronounced differences observed for RPV-Witness and RPV-Media exposures. Among Hispanic participants, increased RPV exposure was also significantly associated with more frequent cannabis use over the past 30 days, across all exposure types (for associations among the total sample, see Figure S1 in the Supplement).

Table 2 presents the unadjusted and adjusted associations between each RPV exposure dimension and substance use outcomes. In bivariate analyses, all RPV dimensions were significantly associated with cannabis and alcohol outcomes, except RPV-Victim in relation to past 30-day and past 12-month cannabis use. Although several associations were attenuated in adjusted models, most remained statistically significant after controlling for sociodemographic factors, everyday discrimination, and psychological distress. RPV-Victim exposure emerged as the most consistent predictor of alcohol use outcomes. In adjusted models it was associated with increased alcohol use in both the past 30 days (IRR, 1.02; 95 % CI, 1.00–1.05) and the past 12 months (OR, 1.04; 95 % CI, 1.01–1.07). For cannabis use, RPV-Witness exposure demonstrated the strongest and most consistent associations, including higher frequency of use in the past 30 days (IRR, 1.02; 95 % CI, 1.00–1.03) and increased odds of past 12-month use (OR, 1.03; 95 % CI, 1.01–1.06).

**Table 2:** Bivariate and Adjusted Effect Size Measures for the Association of Lifetime Exposure to Racism-Based Police Violence and Substance Use Outcomes.

	Model 1 (Unadjusted)^a^	Model 2 (Adjusted)^b^
Alcohol Use Outcomes		
Alcohol Use – Past 30 Days^c^		
RPV – Victim	1.04 (1.02, 1.06)⁎⁎⁎	1.02 (1.00, 1.05)⁎
RPV – Witness	1.03 (1.02, 1.04)⁎⁎⁎	1.02 (1.00, 1.03)⁎
RPV – Media	1.02 (1.01, 1.03)⁎⁎⁎	1.01 (1.00, 1.02)⁎
Alcohol Use – Past 12 Months^d^		
RPV – Victim	1.04 (1.01, 1.06)⁎⁎	1.04 (1.01, 1.07)⁎
RPV – Witness	1.03 (1.02, 1.05)⁎⁎⁎	1.03 (1.01, 1.05)⁎⁎⁎
RPV – Media	1.03 (1.02, 1.04)⁎⁎⁎	1.03 (1.02, 1.05)⁎⁎⁎
Cannabis Use Outcomes		
Cannabis Use – Past 30 Days^c^		
RPV – Victim	1.01 (.99, 1.03)	1.01 (.99, 1.03)
RPV – Witness	1.02 (1.00, 1.03)⁎	1.02 (1.00, 1.03)⁎
RPV – Media	1.01 (1.00, 1.02)⁎⁎	1.01 (1.00, 1.02)⁎⁎
Cannabis Use – Past 12 Months^d^		
RPV – Victim	1.03 (1.00, 1.05)	1.02 (.99, 1.05)
RPV – Witness	1.03 (1.01, 1.06)⁎⁎	1.03 (1.01, 1.06)⁎
RPV – Media	1.02 (1.01, 1.04)⁎⁎	1.02 (1.00, 1.03)⁎⁎

Note. RPV =  racism-based police violence.

a Coefficients and *P* values from *outcome ~ predictor* regression models.

b Adjusted for ethnicity, gender, region, birth in the U.S., Income, Employment, Everyday Discrimination Scale scores, and Psychological Distress scores.

c Effect size measure: Incidence Rate Ratios (IRR).

d Effect size measure: Odds Ratios (OR).

While the point estimates for individual unit increases in RPV exposure appear modest (IRR/OR = 1.01–1.04), these effects become substantial when considered across clinically meaningful ranges of exposure. To illustrate the practical significance of these associations, we calculated the cumulative effects for one standard deviation increases in RPV exposure scores, which represent the difference between individuals with typical low versus high exposure levels (see Table 3).

**Table 3:** Standard Deviation Effects for RPV-Substance Use Associations.

Outcome	RPV Scale	Unit Effect (95 % CI)^a^	1-SD Effect (Black)	1-SD Effect (Hispanic)	% Increase (Black)	% Increase (Hispanic)
Alcohol Use – Past 12 Months^c^	Victim	1.04 (1.01, 1.07)	1.264	1.209	26.4	20.9
Alcohol Use – Past 12 Months^c^	Witness	1.03 (1.01, 1.05)	1.301	1.252	30.1	25.2
Alcohol Use – Past 12 Months^c^	Media	1.03 (1.02, 1.05)	1.496	1.460	49.6	46.0
Alcohol Use – Past 30 Days^b^	Victim	1.02 (1.00, 1.05)	1.125	1.101	12.5	10.1
Alcohol Use – Past 30 Days^b^	Witness	1.02 (1.00, 1.03)	1.193	1.162	19.3	16.2
Alcohol Use – Past 30 Days^b^	Media	1.01 (1.00, 1.02)	1.145	1.136	14.5	13.6
Cannabis Use – Past 12 Months^c^	Victim	1.03 (1.00, 1.05)	1.193	1.154	19.3	15.4
Cannabis Use – Past 12 Months^c^	Witness	1.03 (1.01, 1.06)	1.301	1.252	30.1	25.2
Cannabis Use – Past 12 Months^c^	Media	1.02 (1.00, 1.03)	1.309	1.289	30.9	28.9
Cannabis Use – Past 30 Days^b^	Victim	1.01 (.99, 1.03)	1.061	1.049	6.1	4.9
Cannabis Use – Past 30 Days^b^	Witness	1.02 (1.00, 1.03)	1.193	1.162	19.3	16.2
Cannabis Use – Past 30 Days^b^	Media	1.01 (1.00, 1.02)	1.145	1.136	14.5	13.6

Note. RPV =  racism-based police violence.

⁎ p < .05, ⁎⁎ p < .01, ⁎⁎⁎ p < .001.

a Adjusted for ethnicity, gender, region, birth in the U.S., income, employment, everyday discrimination, and psychological distress.

b Incidence rate ratios from negative binomial regression models reported.

c Odds ratios from ordinal logistic regression models reported.

For alcohol use outcomes, the most pronounced effects were observed for past 12-month consumption. RPV-Media exposure demonstrated the strongest association, with a one standard deviation increase corresponding to a 49.6 % increase in odds for Black or African American participants and a 46 % increase for Hispanic participants (unit OR = 1.03, 95 % CI [1.02, 1.05]). Similar patterns were observed for RPV-Witness and RPV-Victim, with RPV-Victim showing smaller but still meaningful increases. For past 30-day alcohol use, the pattern was similar though somewhat attenuated. RPV-Witness exposure showed the strongest cumulative effect (19.3 % increase for Black or African American participants, 16.2 % for Hispanic participants), followed by RPV-Media exposure (14.5 % and 13.6 % increases, respectively) and RPV-Victim exposure (12.5 % and 10.1 % increases, respectively).

Cannabis use outcomes demonstrated comparable patterns. For past 12-month use, RPV-Media exposure was associated with approximately 31 % increases in odds for both ethnic groups (30.9 % for Black or African American participants, 28.9 % for Hispanic participants), while RPV-Witness exposure showed 30.1 % and 25.2 % increases, respectively. Past 30-day cannabis use showed more modest but still meaningful cumulative effects, with one standard deviation increases in RPV-Witness and RPV-Media exposure corresponding to 14–19 % increases in incidence rates.

In addition, tests of the RPV ×  Ethnicity interaction were conducted to examine whether the associations between the RPV exposure domains and substance use outcomes differed between Black or African American and Hispanic participants. Results indicated that the effects of RPV exposure on substance use outcomes were largely consistent across ethnic groups. Of the twelve interaction models tested (three RPV measures × four substance use outcomes), only one reached statistical significance. The interaction between RPV-Victim exposure and ethnicity for past 30-day alcohol use (F = 4.979, p = .026) revealed that the association between direct victimization and recent alcohol use was stronger among Black or African American participants compared to Hispanic participants. All other interactions (p-values = 0.12–0.96) were non-significant, suggesting that the relationships between RPV exposure and substance use outcomes were comparable across the two ethnic groups for most models examined.

These findings suggest that RPV exposure can operate as a chronic stressor with cumulative health behavior impacts across diverse racial and ethnic groups. Although individual RPV exposure incidents may exert modest associations with substance use, the aggregate burden of repeated exposure appears to elevate substance use risk. The overall consistency of these associations across RPV exposure domains (direct victimization, witnessing, and media exposure) and substance use outcomes (alcohol and cannabis) supports the interpretation that these are meaningful and generalizable public health effects rather than statistical artifacts.

## 4 Discussion

RPV exposure was highly prevalent among this national sample of Black or African American and Hispanic emerging adults, particularly among Black or African American participants, transgender individuals, U.S.-born participants, those with higher incomes, and those employed full-time, demonstrating heightened exposure across multiple RPV domains. These patterns underscore the importance of considering intersectionality when examining the impacts of RPV exposure. Overlapping marginalized identities may compound vulnerability to RPV exposure (Crenshaw et al., 1995, Motley et al., 2024), thereby potentially increasing the likelihood of substance use as a health behavior response to chronic racialized stress (Cruz-Vespa et al., 2024, Gibbons et al., 2010).

Greater RPV exposure was significantly associated with increased alcohol and cannabis use. Specifically, RPV-Victim exposure emerged as the most consistent predictor of alcohol use, while RPV-Witness exposure demonstrated the strongest associations with cannabis use, even after adjusting for sociodemographic characteristics, EDS, and psychological distress. These findings highlight the prevalence and multifaceted associations between RPV exposure and substance use behaviors and suggest that both direct and indirect exposures operate as chronic stressors within a system of racist policing. Rather than framing substance use as a maladaptive coping mechanism, these results suggest that alcohol and cannabis use may represent health behavior responses to cumulative systemic stress, underscoring the potential injurious effects of RPV exposure on well-being.

Our results extend prior research in several important ways. Previous studies have primarily examined RPV exposure among Black or African American participants in geographically localized samples (Motley, Joe, et al., 2023; Motley, Williamson, Pieterse, et al., 2024; Motley, Williamson, and Quinn, 2024). By using a national sample of both Black or African American and Hispanic emerging adults, our study broadens the geographic and demographic scope of prior work, offering a more comprehensive understanding of RPV exposure across multiple ethnic groups. These findings underscore and align with research examining discriminatory events and substance use among diverse populations (Borrell et al., 2007, Glass et al., 2020, Parker et al., 2017, Reyes et al., 2021). For example, Lei et al., (2021)found that experiences of racial and physical discrimination among a nationally representative sample of emerging adults had short-term and cumulative adverse impact on their use of alcohol, cannabis and other illicit drug use, with the associations lasting up to 6 years post-discrimination exposure. Similarly, a study by Jones et al., (2022) found that higher rates of racism-based experiences was a strong predictor of reported substance use in the past month for both alcohol and cannabis among Black or African American male emerging adults.

Beyond these contributions, the present research distinguishes between types of RPV exposure. Most previous studies have emphasized either direct victimization or media exposure (English et al., 2017, Motley and Baidoo, 2023; Motley, Joe, et al., 2023; Motley, Williamson, Pieterse, et al., 2024; Motley, Williamson, and Quinn, 2024), often overlooking the potential distinct impact of witnessing police violence in person. Our results highlight the importance of distinguishing among victimization, witnessing in-person, and media-based exposure, as each represents a unique modality through which individuals may experience perceived racialized policing. Notably, RPV-Witness showed stronger associations with cannabis use than RPV-Media. Although no studies have directly compared substance choice by type of RPV exposure, our findings may reflect differences in coping strategies, perceived threat, and social context. Individuals directly exposed to RPV may experience acute distress and preferentially use alcohol, a socially sanctioned and immediately available depressant, to dampen intense emotions (Cano et al., 2023, Hawn et al., 2020, Johnson et al., 2025). In contrast, those who witness RPV may perceive less personal danger but heightened vicarious stress, making cannabis, a substance often viewed by emerging adults as stress or mood regulating, more appealing (Cruz-Vespa et al., 2024). These patterns are consistent with evidence that both direct and indirect exposure to a perceived racism-based event can differentially influence substance use among ethnic minority populations (Cruz-Vespa et al., 2024, Ebrahimi et al., 2024, Motley et al., 2022), underscoring the need to consider type of exposure when examining potential health behavior outcomes and coping responses.

Notably, we observed a dose-response pattern in substance use across increasing levels of RPV exposure, consistent with prior evidence linking cumulative racialized trauma experiences to adverse health behavior outcomes (Myers et al., 2015, Park et al., 2024). Importantly, the observed relationships between RPV exposure and substance use remained significant after adjusting for both EDS and psychological distress. This suggests that RPV may represent a distinct and particularly deleterious form of structural racism associated with substance use outcomes that are not fully accounted for by measures of non-police involved racism-based experiences or psychological distress. These findings highlight the unique behavioral burden linked to aggressive and perceived RPV practices on marginalized communities.

## 5 Limitations

This study has several limitations that warrant consideration. First, the cross-sectional design precludes any causal inferences regarding the directionality between RPV exposure and substance use behaviors. Longitudinal studies are needed to determine whether RPV exposure precedes increased alcohol and cannabis use or whether substance use may also contribute to the likelihood of RPV exposure. Second, the use of self-reported data introduces the potential for recall bias or social desirability effects, particularly concerning sensitive topics such as substance use and police encounters. Third, although our sample was quota-matched on key demographics, the use of a nonprobability internet panel may limit generalizability to broader populations, especially individuals without reliable internet access or those in institutional settings. Consequently, our findings should be interpreted with caution, as they may not fully represent the diversity of experiences across geographic regions (urban vs. rural), education levels, or other sociodemographic characteristics not included in the quota-matching procedure. Moreover, online panel participation may be skewed toward individuals with greater internet access, more flexible schedules, or different employment or caregiving responsibilities, which could introduce unmeasured biases affecting substance use patterns and RPV exposure.

Fourth, although we adjusted for important confounders, including psychological distress and EDS, residual confounding from unmeasured factors such as community violence or trauma history remains possible. Additionally, while including both Black or African American and Hispanic participants is a strength, the present study could not capture the substantial within-group heterogeneity (e.g., national origin, immigration status) that shapes policing experiences, underscoring the need for future research that disaggregates these populations for a more granular understanding. Finally, while our focus was to explore the relationship between lifetime experiences with RPV exposure and cannabis and alcohol use, the nature of the cross-sectional design did not allow us to account for temporal order and to what extent the use of these substances may have led to RPV exposure. Thus, it is important that future studies account for the possibility of a bidirectional relationship between RPV exposure and substance use by employing longitudinal designs that measure both exposures and substance use behaviors repeatedly over time, enabling stronger tests of temporal sequencing and causal pathways.

## 6 Conclusion

This study provides robust evidence that RPV exposure is widespread and significantly associated with increased alcohol and cannabis use among Black or African American and Hispanic emerging adults in the United States. These findings underscore the need to recognize police violence not only as a civil rights issue but also as a public health concern with potential health behavior consequences (American Public Health Association, 2018, Mattingly et al., 2022). Importantly, the finding that RPV-witnessing exposure was the strongest predictor of cannabis use suggests that interventions cannot be limited to direct victims but must also address the community-wide impact of vicarious exposure. Interventions to reduce substance use disparities should therefore address the collective trauma produced by vicarious RPV exposure through trauma-informed mental health services and community healing initiatives. Moreover, a focus on culturally responsive prevention efforts is critical, particularly among emerging adults of color, as recent research highlights how racial discrimination may heighten the observed national increase in reported use of alcohol, cannabis, and tobacco among adolescents (Rosales et al., 2025).

Building on research on stress, coping, racial trauma, and microaggressions (Carter, 2007, Lazarus and Folkman, 1984, Sue et al., 2007), our findings suggest that alcohol and cannabis use may be understood not as maladaptive but as possible responses to chronic systemic stress. This perspective highlights the need for trauma-informed, culturally responsive prevention and intervention initiatives within community-based organizations to mitigate the impact of RPV exposure and reduce substance use disparities (Barrita et al., 2023, Beeler et al., 2024). Such initiatives can also inform training for law enforcement personnel to increase awareness of racism-based policing practices and to adopt approaches that reduce harm and foster trust in heavily policed communities (Marineau et al., 2025). Future research should employ longitudinal designs to clarify the directional association between RPV exposure and substance use and to identify risk factors that increase vulnerability as well as protective factors that buffer adverse effects. In addition, future studies should examine how socioeconomic status (e.g., income, employment) intersects with the frequency of RPV exposure and associated substance use outcomes. As a result, such studies could inform and refine culturally responsive prevention and intervention efforts aimed at dismantling racism-based policing practices and reducing substance use disparities.

Author Disclosure Statement:

## Statement of authorship

Robert O Motley Jr., Eric Williamson, Melissa McTernan, Christopher P. Salas-Wright: Designed the study. Eric Williamson, Melissa McTernan: Analyzed the data. Robert O Motley Jr: Drafted the original manuscript. All authors (Robert O Motley Jr., Eric Williamson, Melissa McTernan, Sara Beeler, Christopher P. Salas-Wright) reviewed and revised the manuscript and approved the final article. Robert O Motley Jr supervised the study.

## CRediT authorship contribution statement

**Eric Williamson:** Writing – review & editing, Writing – original draft, Methodology, Formal analysis, Conceptualization. **Melissa McTernan:** Writing – review & editing, Methodology, Formal analysis, Conceptualization. **Motley Jr Robert O:** Writing – review & editing, Writing – original draft, Project administration, Methodology, Investigation, Funding acquisition, Data curation, Conceptualization. **Sara Beeler:** Writing – review & editing, Writing – original draft, Conceptualization. **Salas-Wright Christopher P:** Writing – review & editing, Funding acquisition, Conceptualization.

## Funding

This work was supported by the Robert Wood Johnson Foundation Evidence for Action program [5113291]. The content is solely the responsibility of the authors and does not necessarily represent the official views of the Robert Wood Johnson Foundation.

## Declaration of Competing Interest

Author, Robert Motley, PhD declares that he has no conflict of interest.

Author, Eric Williamson, BA declares that he has no conflict of interest.

Author, Melissa McTernan, PhD declares that she has no conflict of interest.

Author, Sara Beeler, PhD, LCSW, MPA declares that she has no conflict of interest.

Author, Christopher P. Salas-Wright, PhD declares that he has no conflict of interest.
