# Supplementary material for: Perceived racism-based police violence and substance use among black and hispanic emerging adults: Evidence from a national sample
Source: Drug Alcohol Depend Rep. 2025 Oct 15;17:100388. doi: 10.1016/j.dadr.2025.100388 (PMC12554227; doi:10.1016/j.dadr.2025.100388)
Supplement: Supplementary file 1 — Supplementary material [file mmc1.docx]

**Figure S1**

*Substance Use Outcomes by Type of Lifetime Exposure to Racism-Based Police Violence*


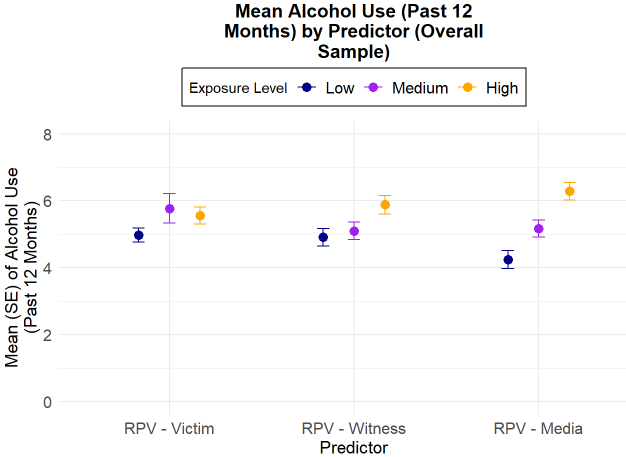


a

a

$\eta^{2}$= .0061

(small effect)

$\eta^{2}$= .0103

(medium effect)

$\eta^{2}$= .0396

(medium effect)


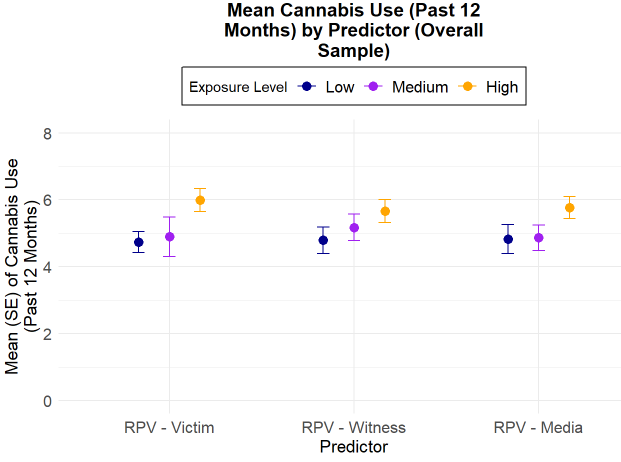


a

a

a

Note. RPV = racism-based police violence

$\eta^{2}$= .0188

(medium effect)

$\eta^{2}$= .0070

(small effect)

$\eta^{2}$= .0110

(medium effect)


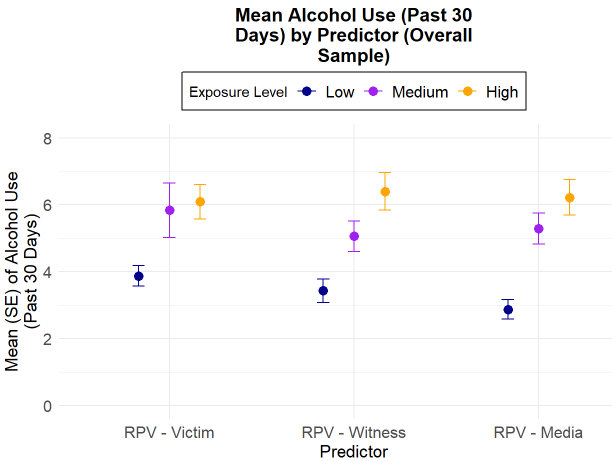


a

a

a

$\eta^{2}$= .0268

(medium effect)

$\eta^{2}$= .0343

(medium effect)

$\eta^{2}$= .0438

(medium effect)


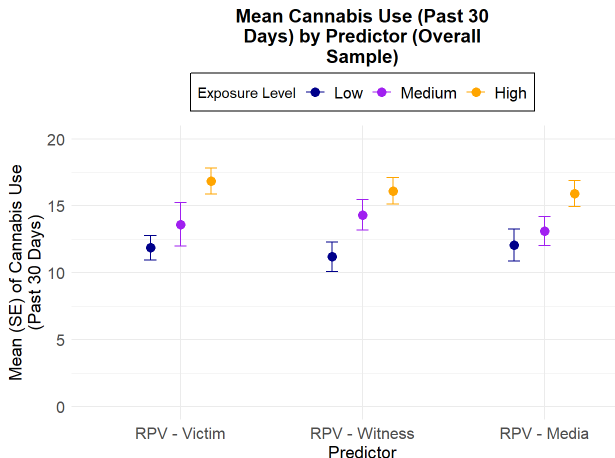


a

$\eta^{2}$= .0179

(medium effect)

$\eta^{2}$= .0267

(medium effect)

$\eta^{2}$= .0340

(medium effect)

Note. RPV = Racism-based police violence

^a^ Significant ANOVA result (p < .05).
