# Supplementary material for: Perceived racism-based police violence and substance use among black and hispanic emerging adults: Evidence from a national sample
Source: Drug Alcohol Depend Rep. 2025 Oct 15;17:100388. doi: 10.1016/j.dadr.2025.100388 (PMC12554227; doi:10.1016/j.dadr.2025.100388)
Supplement: Supplementary file 2 — Supplementary material [file mmc2.docx]

**Appendix A**

**Supplementary Methodology**

In the analysis, participants were categorized into tertiles (low, medium, high) for each RPV exposure type. For the RPV-Victim scale (range: 0-43), tertile cutpoints were 0.1 and 1.1, resulting in 54.8% low exposure (0-0, i.e., no exposure), 12% medium exposure (0.1-1.0), and 33.2% high exposure (1.1-43.0) groups. For the RPV-Witness scale (range: 0-48), tertile cutpoints were 0.1 and 5.1, resulting in 40.1% low exposure (0-0, i.e., no exposure), 29.6% medium exposure (0.1-5.0), and 30.3% high exposure (5.1-48.0) groups. For the RPV-Media scale (range: 0-48), tertile cutpoints were 6.1 and 19.4, resulting in 33.7% low exposure (0-6), 33% medium exposure (6.1-19.3), and 33.3% high exposure (19.4-48.0) groups.

Missing data rates varied across study variables, ranging from 0% for RPV exposure scales to 56.9% for cannabis use measures. All RPV scales (Victim, Witness, Media) and primary demographic variables (ethnicity, region, nativity, income) had complete data. Substance use outcomes exhibited higher missingness, with cannabis measures showing the greatest rates (30-day use: 56.9%, 12-month use: 56.6%), followed by alcohol 30-day use (35.3%) and illicit substance misuse scale (36.2%). Complete case sample sizes for primary analyses ranged from 403 participants (43.1% of total sample) for cannabis use models to 730 participants (78.0%) for alcohol 12-month analyses. Chi-square tests revealed systematic missing data patterns, indicating data were not missing completely at random. Employment status was significantly associated with missingness across multiple substance use variables (χ² = 7.99-34.03, all p < 0.05). Income level showed significant associations with missing alcohol data (χ² = 8.35-20.62, all p < 0.05), while gender was associated with missing cannabis data (χ² = 10.50-10.70, all p < 0.05). Ethnicity demonstrated significant associations with missing alcohol and illicit substance data (χ² = 4.52-6.66, all p < 0.05).

Diagnostic analyses revealed no concerning multicollinearity among predictor variables. Pearson correlations among continuous predictors ranged from 0.244 to 0.645, with the highest correlation observed between RPV-Victim and RPV-Witness scales (r = 0.645). RPV scales showed moderate correlations with the Everyday Discrimination Scale (r = 0.304-0.431), indicating conceptual overlap without problematic multicollinearity. Variance inflation factor (VIF) values across all regression models remained well below concerning thresholds, with maximum VIF = 1.59 (Table A1). Tolerance values ranged from 0.631 to 0.890, all above the 0.20 threshold for multicollinearity. These diagnostic results support the appropriateness of current model specifications and suggest that coefficient estimates should remain stable and interpretable across different outcome measures. The moderate intercorrelations among RPV scales provide evidence for their conceptual distinctiveness while justifying their separate inclusion in regression models.

**Table A1**

*Variance Inflation Factor (VIF) Values For Coefficients Across Models*

| Variable | Alcohol (30 days) | Alcohol (12 months) | Cannabis (30 days) | Cannabis (12 months) |
| --- | --- | --- | --- | --- |
| RPV - Victim | 1.431 | 1.377 | 1.382 | 1.391 |
| RPV - Witness | 1.562 | 1.535 | 1.584 | 1.592 |
| RPV - Media | 1.231 | 1.299 | 1.256 | 1.255 |
| Ethnicity | 1.088 | 1.078 | 1.099 | 1.096 |
| Gender | 1.025 | 1.023 | 1.028 | 1.029 |
| Region | 1.018 | 1.017 | 1.024 | 1.024 |
| Born in the U.S. | 1.039 | 1.040 | 1.026 | 1.026 |
| Income | 1.067 | 1.063 | 1.097 | 1.099 |
| Employment | 1.089 | 1.086 | 1.130 | 1.132 |
| Everyday Discrimination Score | 1.256 | 1.217 | 1.174 | 1.177 |
| Kessler Scale of Psychological Distress | 1.172 | 1.168 | 1.124 | 1.127 |

*Note*. RPV = racism-based police violence; VIF > 5 indicates potential multicollinearity; VIF > 10 indicates serious multicollinearity.
